# Panoramic tongue imaging and deep convolutional machine learning model for diabetes diagnosis in humans

**DOI:** 10.1038/s41598-021-03879-4

**Published:** 2022-01-07

**Authors:** Saritha Balasubramaniyan, Vijay Jeyakumar, Deepa Subramaniam Nachimuthu

**Affiliations:** 1 Department of Electronics and Communication Engineering, Jai Shriram Engineering College, Tiruppur, Tamil Nadu 638 660 India; 2 Department of Biomedical Engineering, Sri Sivasubramaniya Nadar College of Engineering, Chennai, Tamil Nadu 603 110 India; 3 Department of Electrical Engineering, National Institute of Technology Arunachal Pradesh, Yupia, Papum Pare District, Arunachal Pradesh 791112 India

**Keywords:** Biomarkers, Diseases, Health care, Risk factors, Signs and symptoms

## Abstract

Diabetes is a serious metabolic disorder with high rate of prevalence worldwide; the disease has the characteristics of improper secretion of insulin in pancreas that results in high glucose level in blood. The disease is also associated with other complications such as cardiovascular disease, retinopathy, neuropathy and nephropathy. The development of computer aided decision support system is inevitable field of research for disease diagnosis that will assist clinicians for the early prognosis of diabetes and to facilitate necessary treatment at the earliest. In this research study, a Traditional Chinese Medicine based diabetes diagnosis is presented based on analyzing the extracted features of panoramic tongue images such as color, texture, shape, tooth markings and fur. The feature extraction is done by Convolutional Neural Network (CNN)—ResNet 50 architecture, and the classification is performed by the proposed Deep Radial Basis Function Neural Network (RBFNN) algorithm based on auto encoder learning mechanism. The proposed model is simulated in MATLAB environment and evaluated with performance metrics—accuracy, precision, sensitivity, specificity, F1 score, error rate, and receiver operating characteristics (ROC). On comparing with existing models, the proposed CNN based Deep RBFNN machine learning classifier model outperformed with better classification performance and proving its effectiveness.

## Introduction

Diabetes is a worldwide serious metabolic disease that affects well-being of humans by increasing glucose level in blood and it is one of leading causes of death throughout the world. According to the study made by Saeedi et al., it is estimated about 463 million peoples are affected by diabetes in 2019 and the study is extended to identify the possible number of cases that may get affected in 2030 is to be 578 million and by 2045 this may even rise to 700 million^1^. The inability of the body to completely utilize the glucose produced by cells due to improper secretion of insulin in pancreas is diabetes; if the produced insulin is not sufficient to convert the entire glucose into energy then the disease is characterized as Type 2 diabetes. This type of disease commonly prevails among majority of peoples of age group above 40 years but in some cases the beta cells of the pancreas are completely damaged so that the insulin is not produced at all, this type is termed as Type 1 diabetes. The complications of the disease include neuropathy, nephropathy, retinopathy and cardio vascular diseases, Ljubic et al. Therefore, the early diagnosis of the disease and treatment is always a challenging task for Diabetologists concerned^2^.

**Figure 1:**
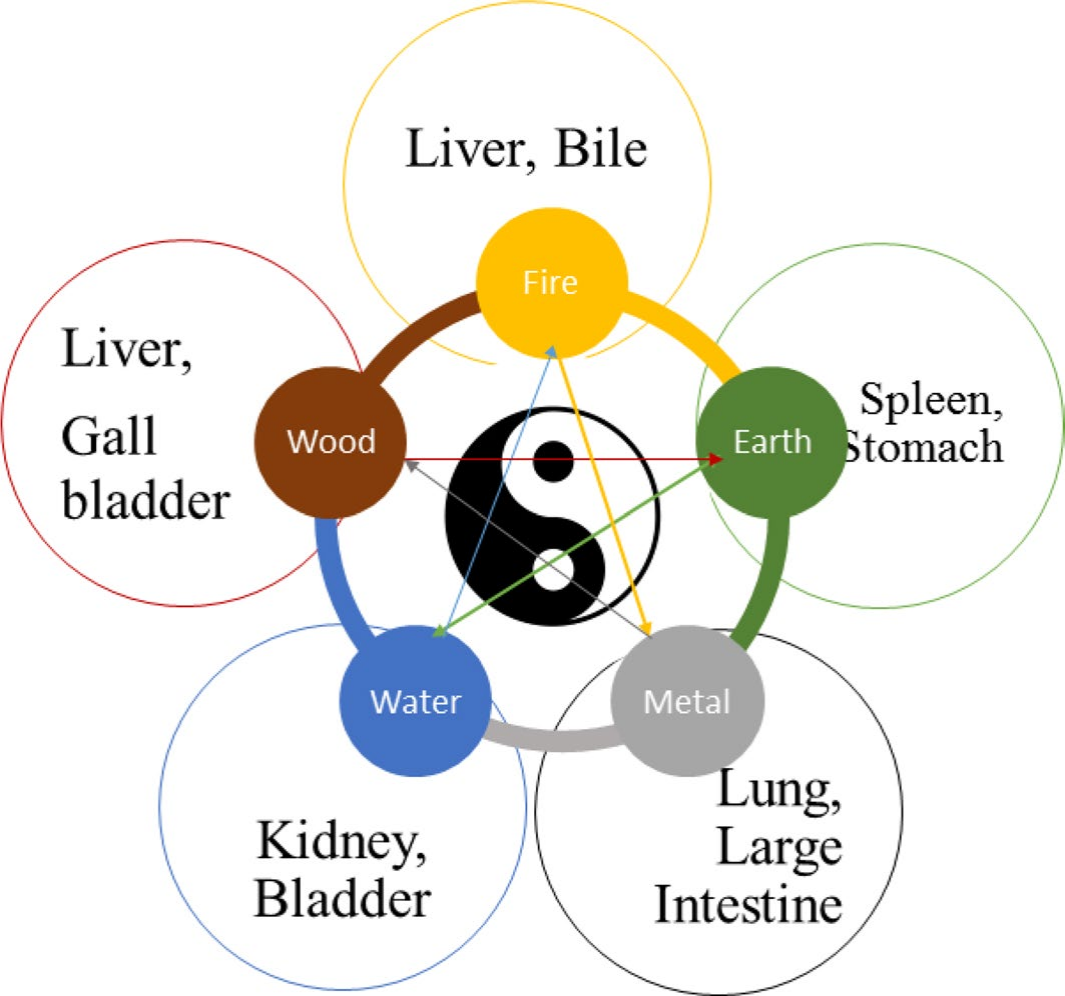
Five elements and Yin-Yang theory.

For the past few years, the Chinese medicine has got great attention towards early prognosis and treatment of diabetes. The diagnosis strategy followed by the Western Medicine (WM) and Traditional Chinese Medicine (TCM) is different from each other, Dong ^3^. TCM follows two basic theories to study the human body, for a healthy wellbeing there should be a perfect balance between five basic elements of universe such as earth, fire, water, metal and wood. All the natural phenomena is categorized into Yin-Yang function, where both Yin and Yang are opposite to each other and they cannot exist without one another as shown in Fig. 1. In TCM the diabetes is caused due to ‘Yin’ deficiency that resulted in frequent drinking and urination, the category of ‘Xiao-Ke disease’. The tongue diagnosis is the main strategy employed in TCM to identify the Yin-Yang disorders, based on which numerous diseases can be diagnosed at its early stage such as breast cancer (Lo et al.), rhumatoid arthiritis (Xie et al.), chronic disease (Sang et al.), lung disease syndrome (Buditjahjanto et al.), liver disease (Cao et al.), spleen and stomach (Bai et al.) and so on^4–9^. The development of high accuracy automatic tongue diagnosis system is always an open field of study, since the human based analysis requires high degree of experience and knowledge for feature interpretation in TCM.

## Existing works of literature

Over the past few years, works have been carried out for various diagnoses employing the features extracted from the tongue images of humans. Few literature works on research of tongue image based diagnosis strategies are presented in this section as follows:

Zhang et al. presented a non-invasive strategy to diagnose non-proliferative diabetic retinopathy (NPDR) based on the tongue features such as colour, texture and geometry with small number of dataset^10^. The tooth marking is the index of *Q*_*i*_ deficiency syndrome diagnosis, the convex hull of tongue is constructed by Graham Algorithm to perform effective tooth marking identification in the work of Li et al.^11^. The statistical distribution characteristics of tongue color and the tongue color gamut was established to identify red spots petechial point for tongue diagnosis purposes^12^. Wang et al. performed tooth marking identification in tongue images by computing the slope of the margin, length and degree of concave regions of tongue^13^.

Shao et al. concentrated on feature of convex region to predict the tooth marking of tongue images^14^. Preshiya presented a diabetes diagnosis based on color image segmentation method. The color of tongue represents the effective working of internal organs such as pancreas, liver, intestines, liver and so on^15^. Kawanabe et al. employed machine learning strategies to perform tongue color based diagnosis strategy based on Japanese Traditional Medicine^16^. For early prognosis of diabetes disease support vector machines (SVM) based strategy was designed with 296 diabetic and 531 non-diabetic patient tongue images and the model reported 83.06% of classification accuracy on employing PCA for feature selection and GA for parameter optimization^17^. The SVM is simple and better classifier model, whereas it has the limitation of large dataset handling issue.

Huo et al. proposed a tongue shape classification model and here the image quality was enhanced by employing Gabor filter, the feature extraction and classification was made by AlexNet CNN technique^18^. The surface of tongue color is an essential feature defining the syndrome and the tongue color extraction was employed for various disease diagnoses including diabetes^19,20^.

Tania et al. studied the research gap in the current works of Automatic Tongue Diagnosis systems^21^. Joshi and Chawan employed machine learning strategies for diabetes diagnosis by using SVM, Logistic regression and artificial neural network methods^22^. The concave regions of tongue was used to perform tooth making identification in most of literatures but in case of perfect dimension the result was inconsistent, Li et al.^23^. So, CNN was employed to identify the deep features then multi-intense classifier was employed over the features to take final decision.

Srividhya and Muthukumaravel performed tongue shape, colour, size and texture based disease classification based on self-organizing map Kohonen Classifier technique^24^. Shen et al. developed a tongue–machine–interface based on passive magnetic localization strategy^25^. Thirunavukkarasu et al. made diabetes classification based on the thermal variations of tongue, the RGB color histogram was employed to extract the features, and the classification was made based on convolutional neural network (CNN) technique^26^. The tooth marking was one of the features associated with diabetes disease, Wang et al. developed a ResNet 34 CNN strategy for tooth marking identification in tongue images^27^.

Naveed employed fractional order Darwinian particle swarm optimization algorithm to classify diabetes based on tongue features such as color and texture^28^. Wu et al. proposed a CNN based diabetes diagnosis strategy to perform image classification, small number of training data was utilized with a kind of transfer learning strategy adopted to improve the learning speed^29^. The background of diabetes and the disease complication was reviewed, the overview of decision support models based on machine learning strategies was presented in Lim et al.^30^.

Vijayalakshmi et al. utilized features such as color, texture, geometry of tongue for diabetes diagnosis based on SVM-CNN strategy^31^. To perform an effective tongue diagnosis, a combination of histogram of oriented gradients (HOG) and the support vector machine (SVM) was developed in the study made by Yuan and Liao, where the tongue coating was extracted by k-means segmentation strategy^32^. A Multi-Task Joint learning (MTL) for tongue segmentation and classification based on deep learning technique (Xu et al.), here the UNET and Discriminative Filter Learning DFL was fused with MTL to improve the classification accuracy^33^.

Tang et al. presented a tooth marked tongue regions based on deep learning strategy, here a two-stage methodology based on cascaded CNN to identify the tongue region and tongue land marks^34^. The tongue color and texture was employed to perform diabetes classification by Kernel Ensemble Classification (KEC) by Selvarani and Suresh ^35^.

Zou et al. has elucidated the various machine learning techniques employed for detecting diabetes mellitus^36^. Mujumdar and Vaidehi employed pipeline based AdaBoost classifier, Gradient Boost classifier and Random forest classifier for predicting diabetes mellitus with the considered datasets^37^. Peng et al. demonstrated the clinical utilities to rapidly stratify diabetes subjects based on their oxidative status in conjunction to the traditional glycemic level to improve the patient stratification and thus the overall outcome of clinical diabetes care and management^38^. Pen et al. developed a new methodology for rapid, label-free molecular phenotyping of biological fluids (e.g., blood) by exploiting the recent advances in fast and highly efficient multidimensional inverse Laplace decomposition technique. Machine learning techniques were introduced to transform the NMR correlational map into user-friendly information for point-of-care disease diagnostic and monitoring^39^.

On investigating the existing works of literatures, numerous models were proposed in general for disease diagnosis and only few are available in the field of diabetes diagnosis. The studies mostly employed few dataset for training the model that affects the model performance and also they highly concentrated on tongue colour and tooth markings as significant features. Based on the review made on all the existing works of this application area, the various limitations inferred includes:

- Few methods are not applicable for large volumes of data^15–20^.
- Difficult to handle increased data dimension^24^.
- Inappropriate extraction of prominent features^12,13^
- Increased computational complexity^22^
- Delayed convergence^26–30^
- Minimal features are employed and hence classification accuracy was not guaranteed^31^.
- Premature convergence with the occurrences of global and local minima problems^14^
- Inconsistent attainment of results^23^

To address the above said limitation the proposed model investigated on various features such as colour, texture, tooth markings, fur colour, fur thickness and so on and developed appropriate hybrid neural network models to diagnose diabetic. The hybrid approach in this research study employs convolutional neural network model for effective feature extraction and a novel deep learning based radial basis function neural model for better classification of diagnosing diabetics with tongue image features.

The novelty in the paper includes the development of new deep radial basis function neural network model with the Gaussian activation function and enabling in the diagnosis of diabetes mellitus. The developed deep convolutional based radial basis function is modelled in the ResNet 50 being the feature extractor and the new deep RBFNN to be the classifier. Originally, the deep convolutional models themselves modelled to be feature extractor and classifier and in this paper, the effective features of the radial basis function to act as a normal density function is brought out and led in the development of the deep radial basis function neural network model. Additionally, the modelled deep RBFNN is optimized for its deep radial basis layers with 6 layers as shown in Fig. 3 and the deep hidden neurons equal to the number of input neurons in the input layer. Generally, TCM was operated for its prediction applications with simple neural models, but in this paper the novelty is depicted by the utilization of deep convolutional feature extractor and deep radial basis classifier along with the TCM for the most effective detection and classification of diabetes mellitus.

The rest of the article is framed as follows: An overview of Xiao-Ke disease is presented in “Xiao-Ke disease (diabetes mellitus)—an overview”, the novel methodology for better classification is discussed in “Methods and materials”, the proposed Deep RBFNN model is elucidated in “TCM based diabetes diagnosis in humans with proposed technique”, results and discussion made to prove validity of proposed technique is provided in “Results and discussions”, and finally “Conclusion” presents the conclusion of this research study based on the investigations done.

## Xiao-Ke disease (diabetes mellitus)—an overview

In Traditional Chinese Medicine (TCM), a disease is diagnosed based on the harmony that exist between five basic elements such as wood, fire, earth, metal and water, where each element are connected with specific organ of the human body. The functions of the organs, the emotional states, and symptoms of disease can be analyzed based on Yin-Yang theory. The ‘Yin’ is the dark portion of Yin-Yang that shows the cold, negativity, illness, slow, inward, and passive. The ‘Yang’ is the bright portion that shows brightness, high strength, positivity, heat, excitement and so on. In ‘Yin’ there is always small portion of ‘Yang’ and ‘Yang’ is always associated with small portion of ‘Yin’.

The diabetes is categorized as Xiao-Ke syndrome in TCM, which means “Wasting” and “Thirsting”, the disease gets associated with the organs of lungs, stomach and the kidneys. Traditionally the Xiao-Ke is classified as three types—the lower, middle and the upper which are characterized by the symptoms of excessive urination, excessive hunger and excessive thirst respectively. If a patient has symptom of excessive urination, it can be described as the ‘Yin’ deficiency of lung and kidney that produces high internal heat and utilizes high body fluids so the patient is reported with excess thirsting.

In TCM the tongue is considered as index of all organs, it is connected through meridians to the internal organs of the body. The tongue is the visual indicator of the body functions (Gabhale et al.) and its appearance reflect the balance that exist between the five elements, so the one who perform diagnosis can understand the physical and mental health of the patient based on the tongue features such as color, shape, coating, texture, tooth markings, and so on^40^. Numerous studies has been carried out on identifying the features for tongue based diabetes diagnosis^41^, Hsu et al. detailed that the yellow fur, thick fur and the bluish tongue are the features associated with high prevalence of diabetes and tooth marked tongue identification by employing deep CNN model was done by Wang et al., tongue thermography as diagnosing tool for type 2 diabetes, works concentrated on tooth marking and color identification process as well^26,27^.

**Figure 2:**
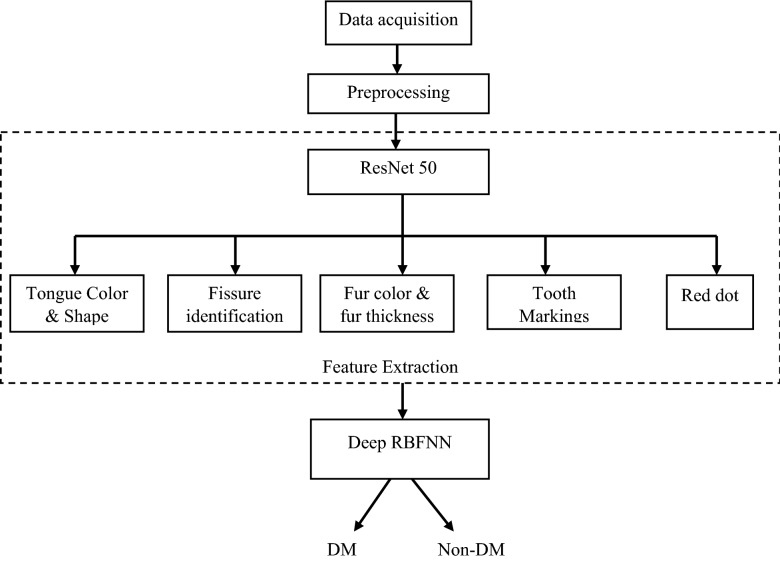
Block diagram of the proposed diabetes diagnosis research study with panoramic tongue imaging technology.

In this research study, the tongue features such as tooth mark, fur color, fur thickness, tongue shape, saliva, tongue color and red dot are extracted by employing deep convolutional neural network, and the extracted features are fed into deep Radial Basis Neural model to flag the tongue as belonging to normal class or affected class (diabetes mellitus (DM) or non-diabetes mellitus (non-DM)). Figure 2 presents the block diagram representation of the proposed diabetes diagnosis research study.

## Methods and materials

In this research study, a novel hybrid convolutional neural network framework with deep radial basis function neural network model is developed with the prominent features from the tongue images collected from hospitals. This section details the methods employed and data used for performing effective diabetic diagnosis by extracting the most significant features from the considered image datasets.

### Panoramic tongue image acquisition

The collected tongue image datasets reflects the actual body condition and it does not get altered by other external factors, the color of the tongue, the thickness, and the surface of the tongue can be altered by certain food beverages such as coffee, tea, and ice cream, chocolate and so on. The tongue color is considered as one of the main feature for disease diagnosis, if the participant consumes fruit like blue berries the tongue color will turn blue, consuming spicy food will change the color to bright red, which will affect the diagnosis result. Also, the brushing process removes the coating of tongue and it will appear to be thin, which will affect the diagnosing accuracy, moreover the thickness of the tongue gets thinner as the day elapses. So, considering these factors the participants are requested not to brush or consume any food before their tongue image is captured and the image is captured early in the morning before the participant have their breakfast.

Mild red color, considerably wet, covered with thin white layer are the nature of normal tongue, whereas it changes for various seasons. The white coating slightly becomes yellow due to hot climatic condition in summer and the coating becomes thin and dry during autumn, becomes highly moist during winter season. The yellow coating and dryness are the features representing the disease, so there is possibility to label the normal patient as a diabetic patient which is highly dangerous. In the existing works of literature there is no study available on accounting the seasonal changes affecting the tongue features.

On accounting the above mentioned significance; in the proposed research study one year dataset is collected in the diabetic care unit of Kalpana Hospitals, Coimbatore, India, using a high quality panoramic image acquisition module. The data collection is made in four phases, in the month of December—January the first phase of data collection is made and about 530 participants are participated among them 272 are diabetic patients and 258 were non-diabetic patients. The second phase of data collection made in the month of March–May about 750 patient images were collected and among them 483 patients were diabetic and 267 were non-diabetic patients. In the month of June–September the third phase of data collection is made with 723 of diabetic patient record and 339 of non-diabetic patient images. The final phase of data collection is made in the month of October with 180 images of diabetic record and 153 of non-diabetic patient images.

The proposed research study aims to diagnose type 2 diabetes so the patients of type 1, Gestational diabetes are not included in the study. The database of 1658 diabetic patient images with 1017 non-diabetic patients records are segregated onto 70% (1161-DM and 712 of non DM) for training the model and 30% (497-DM and 305 non DM) as independent dataset to evaluate the performance of the proposed diagnosis model.

### Developed ResNet-50 machine learning architecture for diagnostic study

Basically, the deep learning network is difficult to train because of vanishing gradient issues, when the network grows deeper the accuracy gets saturated. On considering this limitation a residual network is employed in this proposed research study. The residual network employs a concept of skip connection strategy; where the original input is added to the loss function at output through skip connection so that the vanishing gradient issue gets addressed. The shortcut connections optimize the ResNets by reducing the training error of the conventional stacked connections by the formulation of $Y = F(X) + X$ at the output side. The basic objective of residual network is making the residue *F(X)* tend to zero, so that the output will be equal to the input at the output end.

**Figure 3:**
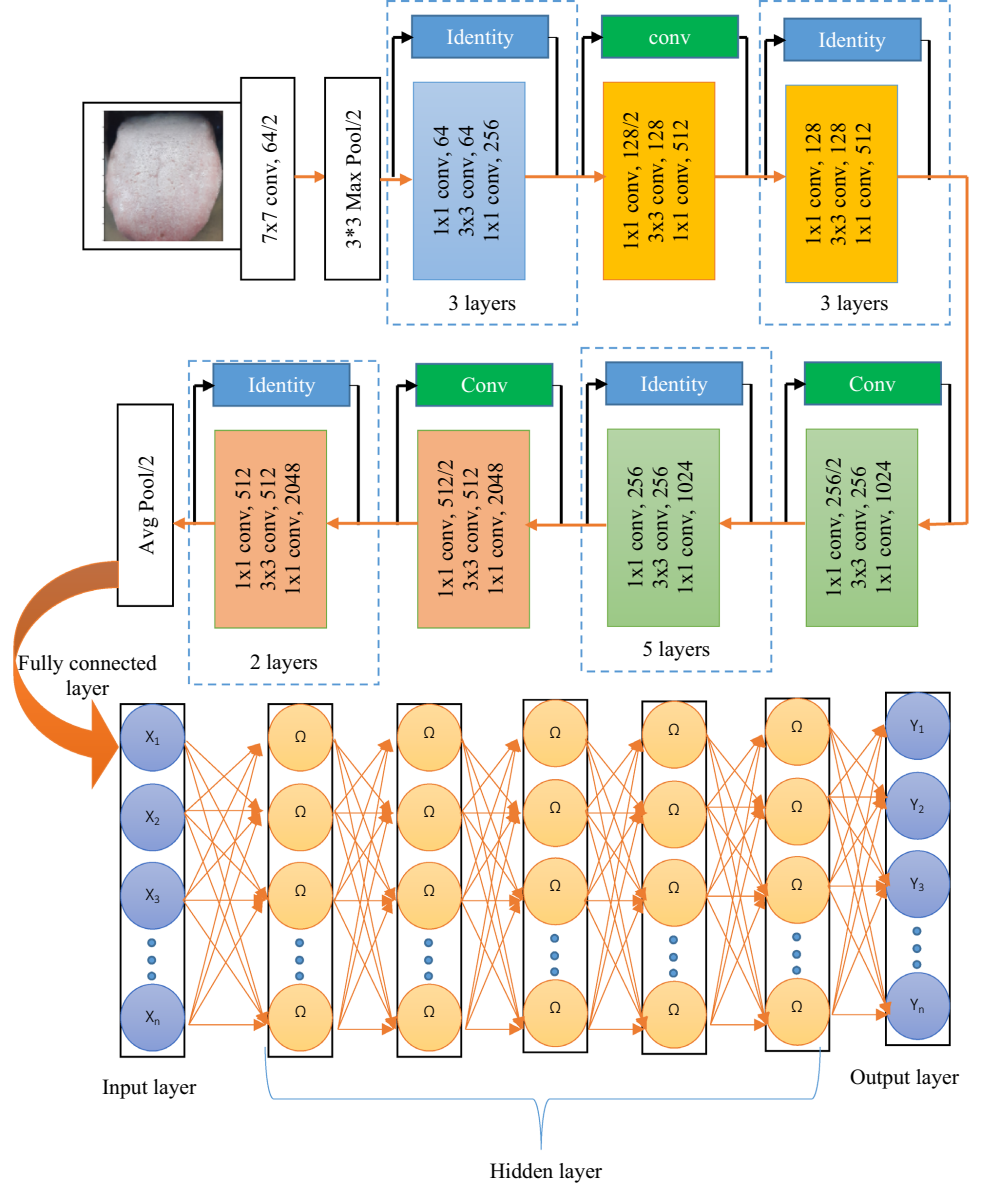
Machine learning architecture of deep CNN-RBFNN for tongue diagnosis.

The architecture of the ResNet-50 employed in this research study is presented in Fig. 3. The input image is fed into the first layer where the filter size is 7 × 7 and number of filters is 64 with stride value of 2 and padding of 3, the max pool is employed for down sampling with the stride of 2. Each of Relu block in ResNet-50 has three feature layers, the skip connection is employed by two ways based on the size of *F(X)* and *X*. The input is directly connected to the output if the size of *F(X)* and *X* are same else a convolution is made in the skip connection to match the size of the input and the functional output if they are varied. The fully connected layer retains the complete feature of the image without any loss, this feature vector is seeded as input for deep radial basis function network to classify the vectors as a diseased one or not.

### Multilayer RBFNN Architecture for panoramic tongue image classification

Neural network forms the base of deep learning where the algorithms are based on the learning behaviour of human brain. The network is trained with set of input–output patterns and the model is tested with new pattern of input and the corresponding response is evaluated. The framework of a neural network model has input and output layer with one or more hidden layers based the network architecture and based on the problem requirement. Deep learning architecture mimics the structure of human brain where numerous neurons are interconnected together to take a specific decision. In this research work, a multilayer Radial Basis Function Neural Network (RBFNN) model with auto-encoder learning mechanism is implemented to learn the features with better generalization ability. The RBFNN is a single layer feed forward neural network like multilayer perceptron and was initially developed by Craddock and Warwick (1996)^42^.

The centre identification in multilayer RBFNN model is not an easy task during learning process, so the k-means clustering algorithm is employed to identify the center. The functional connection between the layers are represented as,

1 $$ f(x) = \sum\limits_{i = 1}^{N} {c_{i} \varphi (\left\| {x_{j} w - r_{i} } \right\|^{2} )} $$

Equation (1) is similar to that of traditional RBFNN model whereas, in multilayer RBFNN a norm function is introduced to locate the position of data points in the high dimensional space. 

Deep auto-encoder based learning mechanism is implemented over layer by layer of hidden neurons, and the data is decoded so that the underlying significant features are reflected at output layer to present specific classes with better and effective accuracy rate. The conventional gradient decent learning rule is employed to update the weight vectors, the objective of the algorithm is to reduce the reconstruction error between the encoded and decoded data. The derivative of error function is back propagated from output layer to the hidden layer based on the error function and then the weight vectors are updated.

The weight vectors between the input and the hidden layers are depicted as $w_{ij}$ and the weight vectors between the hidden and the output layers are presented as $v_{jk}$, the error gradient between the input and the hidden layer is presented as $\delta_{i}$ and $\delta_{k}$; these indicates the error gradient between the hidden and the output layer. The net input is calculated between the input and hidden layer as,

2 $$ z_{ - inj} = \sum\limits_{i = 1}^{n} {x_{i} w_{ij} + b} $$

The net input between the hidden and the output layer is presented as

3 $$ y_{ - ink} = \sum\limits_{j = 1}^{n} {z_{j} v_{jk} + b} $$

The error between the actual and the network output is represented as *E*, the error gradient and is estimated as,

4 $$ E = \frac{1}{2}\left( {\sum\limits_{i = 1}^{n} {y_{t\arg et} - y_{predicted} } } \right)^{2} $$

5 $$ \frac{{\partial E}}{{\partial v_{{jk}} }} = \frac{\partial }{{\partial v_{{jk}} }}\left( {\frac{1}{2}\left( {\sum\limits_{{i = 1}}^{n} {y_{{target}}  - y_{{predicted}} } } \right)^{2} } \right) $$

6 $$ = \frac{1}{2}\frac{\partial }{{\partial v_{jk} }}(y_{t\arg et} - f(y_{ - ink} ))^{2} $$

7 $$ = - [y_{t\arg et} - y_{predicted} ]f^{\prime}(y_{ - ink} )\frac{\partial }{{\partial v_{jk} }}(y_{ - ink} ) $$

8 $$ = - [y_{t\arg et} - y_{predicted} ]f^{\prime}(y_{ - ink} )z_{j} $$

9 $$ \delta_{k} = - [y_{t\arg et} - y_{prediced} ]f^{\prime}(y_{ - ink} ) $$

10 $$ \frac{\partial E}{{\partial w_{ij} }} = - \sum\limits_{k} {[y_{t\arg et} - y_{predicted} ]} \frac{\partial }{\partial wij}(y_{k} ) $$

11 $$ = - \sum\limits_{k} {[y_{t\arg et} - y_{predicted} ]f^{\prime}(y_{ - ink} )\frac{\partial }{{\partial v_{jk} }}(y_{ - ink} )} $$

12 $$ = - \sum\limits_{k} {\delta_{k} v_{jk} \frac{\partial }{{\partial v_{jk} }}(z_{j} )} $$

13 $$ = - \sum\limits_{k} {\delta_{k} v_{jk} f^{^{\prime}} (z_{inj} )(x_{i} )} $$

14 $$ \delta_{j} = \sum\limits_{k} {\delta_{k} v_{jk} f^{^{\prime}} (z_{inj} )} $$

The change in weight vectors are presented as,

15 $$ \Delta w_{ij} = - \alpha \frac{\partial E}{{\partial w_{ij} }} $$

16 $$ \Delta w_{ij} = \alpha \delta_{k} x_{i} $$

17 $$ \Delta v_{jk} = - \alpha \frac{\partial E}{{\partial v_{jk} }} $$

18 $$ \Delta v_{ij} = \alpha \delta_{j} z_{j} $$

In Eqs. (15) through (18), ‘α’ denotes the learning rate , ‘$\Delta w$’ is the change in weight vector between input and hidden layers and ‘$\Delta v$’ is the change in weight value between the hidden and the output layers, the new weight vectors are updated as,

19 $$ w_{ij} (t + 1) = w_{ij} (t) + \Delta w_{ij} $$

20 $$ v_{jk} (t + 1) = v_{jk} (t) + \Delta v_{jk} $$

The nonlinear transformation within deep layers is made by encoder and decoder based pre- training technique, and the output of one layer forms the input for the next layer. The encoder employs encoding function over the incoming dataset and converts that into coded form, whereas the decoder at output side decodes the data into its original form on utilizing decoding function. On fine-tuning the reconstruction error, the ‘loss function’ is estimated to handle the variation between the decoded and the encoded data. The encoding and decoding function for the given dataset is presented as,

21 $$ encoded\_data = f_{\varphi } \left( x \right) $$

The reconstructed dataset is presented as,

22 $$ \hat{x}^{{}} = g_{\varphi } \left( {encoded\_data} \right) $$

‘$f_{\varphi }$’ is the encoding function and ‘$g_{\varphi }$’ is the decoding function.

The encoding is made layer by layer, the encoded data of first layer is fed as input for second layer and likewise the data which is encoded at last hidden layer is employed to find output at output layer. The error between the encoded and decoded data is given by,

23 $$ E_{error} = \left\| {x - \hat{x}} \right\|^{2} $$

Deep learning neural model in this research study is trained to minimize the loss function, which is the objective function and is presented as,

24 $$ \delta_{error} (x,\hat{x}) = \frac{1}{N}\sum\limits_{i = 1}^{N} {E_{error} \left( {x^{i} ,g_{{\varphi^{^{\prime}} }} \left( {f_{\varphi } \left( {x^{i} } \right)} \right)} \right)} $$

The next step of pre-training is the fine tuning by error back propagation, for the given input data the output at output layer is given by,

25 $$ y_{output} = f_{{\varphi_{N + 1} }} (enocoded\_data^{N} ), $$

where, the Nth encode vector at the last hidden layer is employed to present the final output of the trained network model.

Radial basis functions that are employed as activation function in the designed new deep radial basis function neural network model are radially symmetric functions that get shifted by points in multidimensional Euclidean space and then are linearly combined so that they result in data-dependent approximation spaces. The applicability of the RBFNN with varied design of deep learning architecture increased the classification results by decreasing the error rate with its best generalization ability, strong tolerance to the input signals and better learning ability for image based data input signals presented to the network. The architecture of the deep learning model with the encoding and decoding with the fault tolerant radial basis function tends to make the neural network converge with better classification results. The generalization and learning capability of the radially symmetric radial basis function being used as the activation function in the deep learning model evolves better output from each of the deep learning layers compared to other activation functions like tangential functions, sigmoid functions, and other exponential functions. Hence this RBFNN with its radially symmetric activation function has attained better classification results for the detection of tongue image datasets.

## TCM based diabetes diagnosis in humans with proposed technique

In tongue image based diagnosing procedure the color of tongue should not be affected by image capturing system, it should reflect all colours without any change from the actual image. The collected image is preprocessed to balance the light illumination and the brightness of each image by means of image enhancement and noise removal techniques. The image quality is enhanced by employing Gabor filter algorithm. All the images are calibrated for color correction by histogram equalization technique and the tongue portion is alone segmented, further the motion blur is avoided to improve the texture quality of the images.

**Figure 4:**
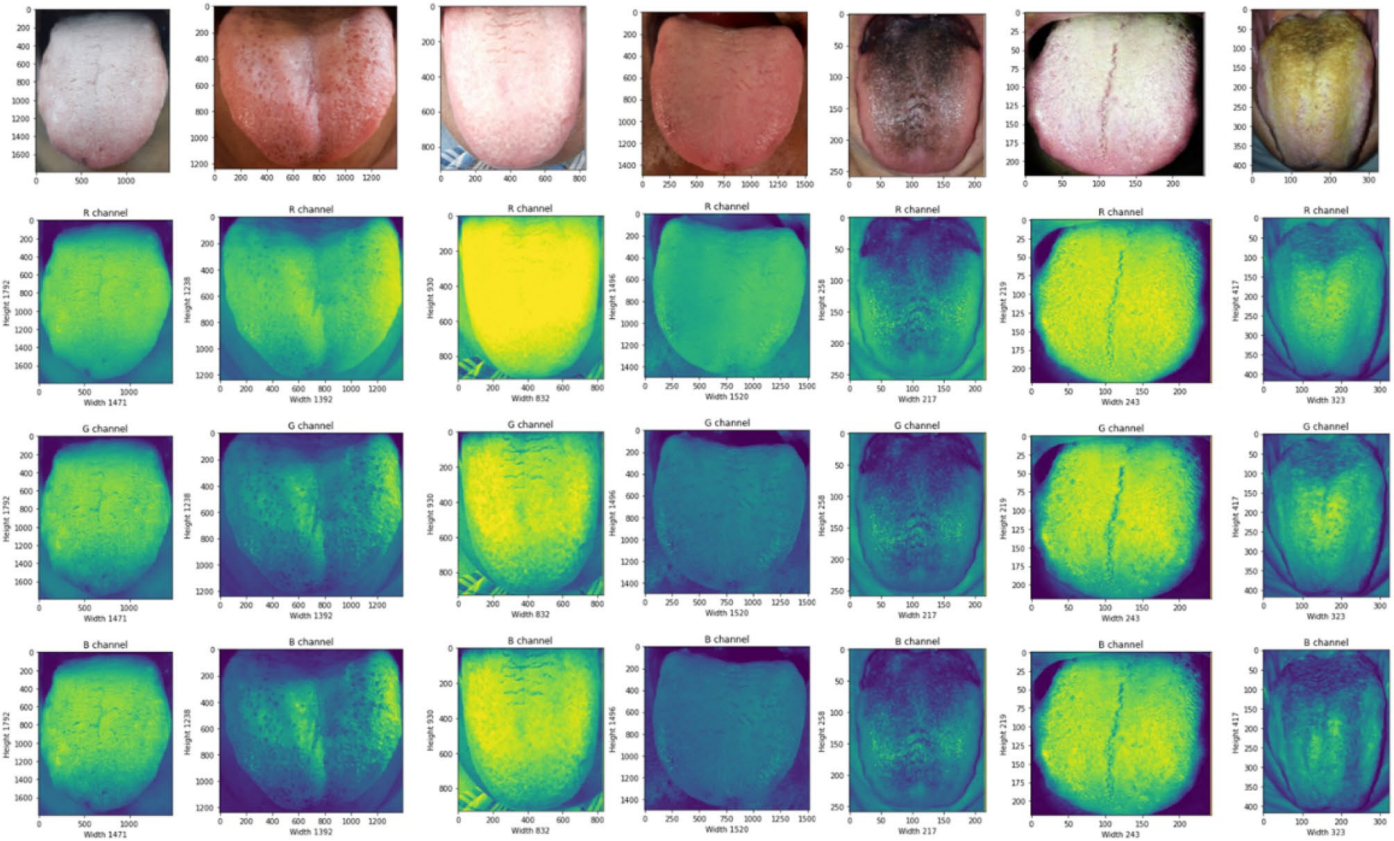
RGB channels for few sample of panoramic tongue images.

**Figure 5:**
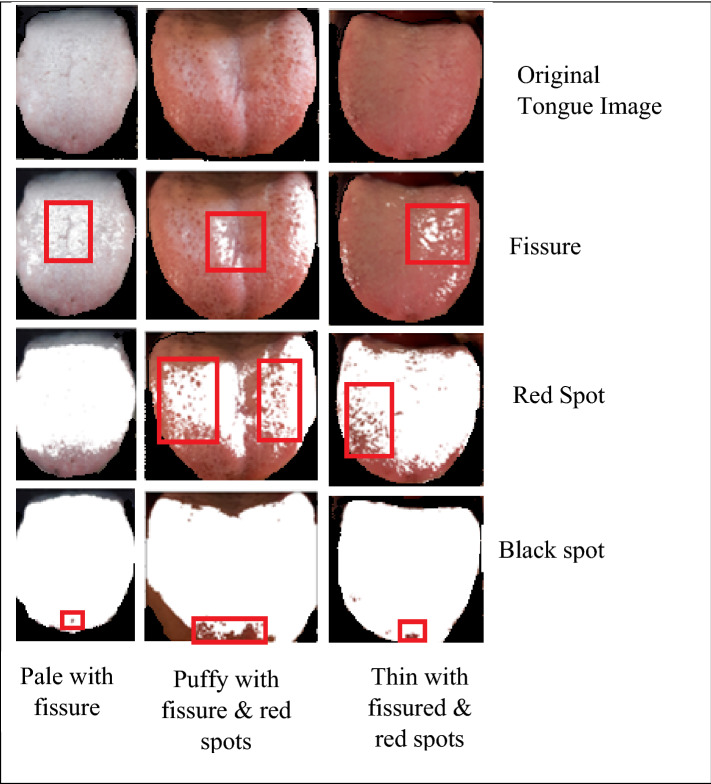
Extracted features of tongue image samples.

The feature extraction is the next step to image pre-processing, numerous feature extraction strategies are available in literatures, but in this research study convolutional neural network (CNN) is considered because during image flattening the information may get lost in conventional techniques whereas the convolutional network model retains the actual information without any loss, and it learn the features by itself. The 2D image is processed as 3D matrix with RGB color channel as depth as shown in Fig. 4. The collected data samples are labeled by TCM experts based on tongue color, texture, tooth markings, fur color and thickness, the features associated with the patient records and are presented in Table 1. The extracted features of the tongue data samples such as tongue color, red spots, black spots, Fissure, dry tongue, tooth markings, saliva, size, and fur are shown in Fig. 5.

**Table 1:** Features associated with patient records.

Features	Types	Number of data samples	Features	Types	Number of data samples
Tongue body	Medium	1332	Teeth markings	Yes	1662
	Enlarged	915		No	1013
	Small	428	Fur color	White	1203
Tongue color	Mild red	1400		Black	201
	Red	677		Yellow	996
	Pale	375	Saliva	Normal	1884
	Bluish	223		Dry	518
Tongue surface	Red spots	1489		Wet	273
	Black spots	1044	Fur thickness	Thin	852
	Fissures	1038		Thick	1606
	Petechiae	205		No fur	217

### Metrics employed for diabetic diagnosis model

To evaluate the effectiveness of the proposed diagnosis model, the performance metrics -accuracy, precision, sensitivity, specificity, F1 score and error rate are evaluated. The accuracy represents how closely the true predictions are made by the proposed classifier, whereas in medical decision making models in addition to accuracy, it is necessary to analyse how accurately the true positive and true negative predictions are made. Because, the false prediction is highly dangerous in the field of diagnosis, so in addition to accuracy, True Positive Rate and False Positive Rate are employed to illustrate the effectiveness of the proposed diagnosis strategy.

#### Accuracy

Accuracy highlights the true positive and negative predictions of the model, the ratio between number of true positive and negative prediction to the total number of all samples is the accuracy.

26 $$ Accuracy = \,\frac{TP + TN}{{TP + TN + FP + FN}} $$

#### Precision

Precision is a measure of how accurate the model performs positive prediction, the ratio between the correct positive predictions and the total number of positive prediction is the precision.

27 $$ \Pr ecision = \frac{TP}{{TP + FP}} $$

#### Recall or sensitivity

Sensitivity reflects what percent of true positive is presented as positive; mathematically it is expressed as the ratio between the correct positive predictions and the sum of correct positive and false negative predictions. The higher the recall the higher the performance of the model.

28 $$ Sensitivity = \frac{TP}{{TP + FN}} $$

#### Specificity

The measure that demonstrates what percent of true negative is identified as negative class by the classification module, the higher the specificity higher the model performance.

29 $$ Specificity = \frac{TN}{{TN + FP}} $$

#### F1 score

It ranges from 0 to 1, when F1 score increases the performance became better. The harmony between the precision and recall is represented by the F1 score.

30 $$ F1 = 2*\frac{precision*recall}{{precision + recall}} $$

#### Error rate

Error rate is defined as ratio between the number of all the false predictions and the total number of samples used in the model. The higher the error rate, then lower is the performance of the model.

31 $$ Error\,rate = \frac{FN + FP}{{TP + TN + FP + FN}} $$

where, *TP* represents the True Positive, FP—False Positive, TN—True Negative, and FN—False Negative. The metric results are evaluated based on the confusion matrix framed in Table 2.

**Table 2:** Confusion matrix framework for diabetes diagnosis.

Actual class	Predicted class True outcome: diabetes identified	
	P (diabetes)	N (non-diabetes)
P (diabetes)	TP (diabetes patients identified as diabetes)	FP (diabetes patients identified as non diabetes)
N (non-diabetes)	FN (non-patients identified as diabetes)	TN (non-diabetes patients identified as non-diabetes)

### Ethics approval and consent to participate

Necessary ethical standard was maintained in this research study. All methods were carried out in accordance with relevant guidelines and regulations of Medical Council of India.

All experimental protocols were approved by a Kalpana Hospitals, Coimbatore. Consent was obtained from all subjects with the approval from Kalpana Hospital Authorities. “Written Informed Consent” has been obtained from participants for whom tongue image datasets were attained. The methods were carried out based on the norms and guidelines of Medical Council of India (MCI). Board of Council members (BCM) of the Kalpana Hospital is the ethics committee that approved the study.

## Results and discussions

Hybrid convolutional neural network with Deep RBFNN model is designed and simulated in this research study for effective diabetes diagnosis and is performed in MATLAB R2019a environment in a Intel Quad Core i7 processor with 3.9 GHz processor speed, 16 GB RAM on 64 bit operating system and with NVIDIA MX250 2 GB Graphics features. The input dataset is preprocessed by employing Gabor filter, the color calibration is done with histogram equalization algorithm. The tongue segment is extracted from the original images and fed into ResNet-50 for feature extraction. The features such as the tongue size, shape, fissure, saliva, red spots and black spots are extracted and presented in Fig. 5. Table 3 lists the parametric values of the proposed deep RBFNN model designed for classifying diabetes mellitus.

**Table 3:** Parameters of the proposed deep neural model.

Parameters	Parametric values
Learning rate	0.2
Number of epochs	Until the network settles at convergence criterion
Convergence criterion	10^–6^
Loss function (mean square error)	$\sum\limits_{t = 1}^{n} {\left( {y_{t} - O{}_{t}} \right)^{2} }$
Learning rule	Gradient Descent learning rule
Optimizer	Ant colony optimizer
Activation function	Gaussian function
Number of deep radial basis layers	5
Batch size	40 samples

**Figure 6:**
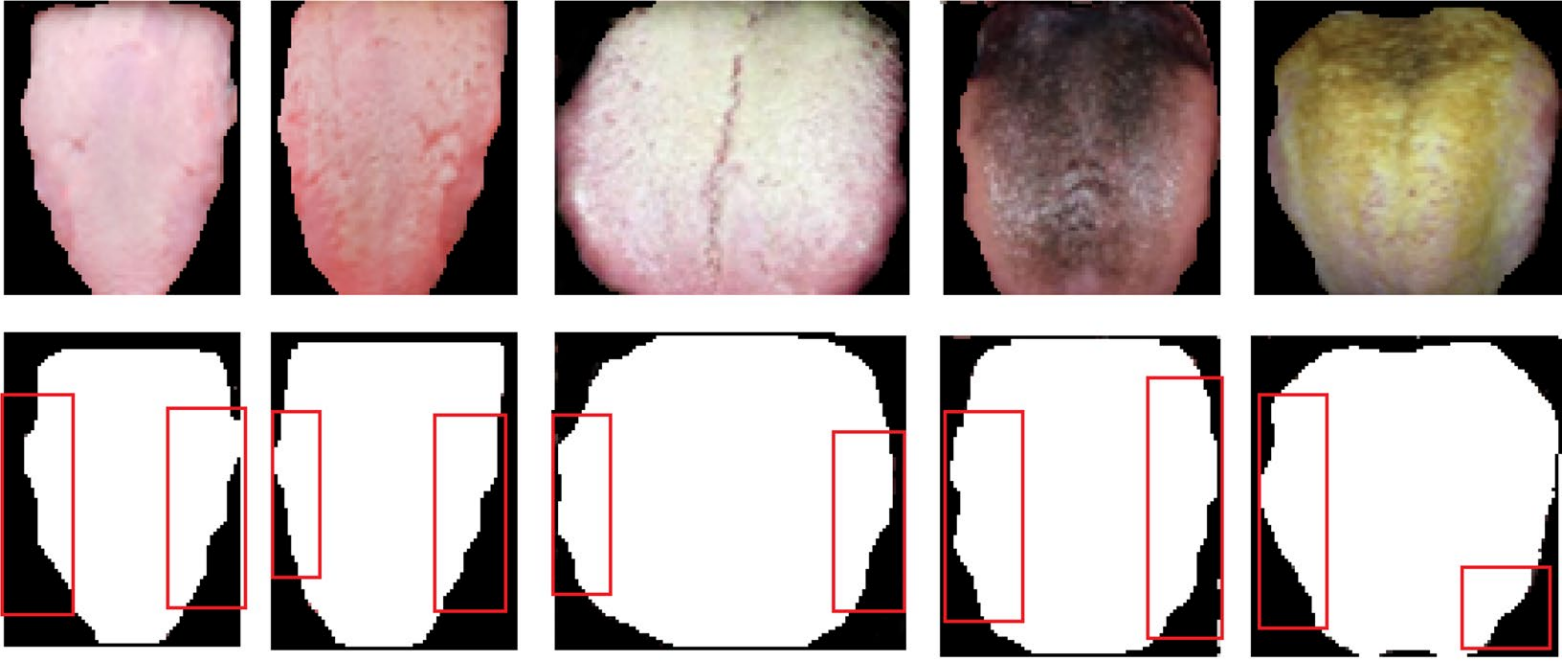
Tooth markings of tongue images.

The color of the tongue indicates the blood and *Qi* (Energy) flow through the body, if the tongue appears to be red then it indicates excess flow of heat, when it is pale then it represents deficiency of heat and deficiency of blood, hence this imbalance of energy causes problems in intestine and pancreas. The shape of the tongue also reflects the working of digestive and pancreas, normally the shape will neither be thin nor be thick, and the characteristics such as swollen (larger and thicker) or puffy tongue indicates the Qi and excess fluid accumulation. The swollen tongue are usually associated with tooth markings that shows the Yang deficiency, the tooth markings for sample of dataset is presented in Fig. 6.

**Figure 7:**
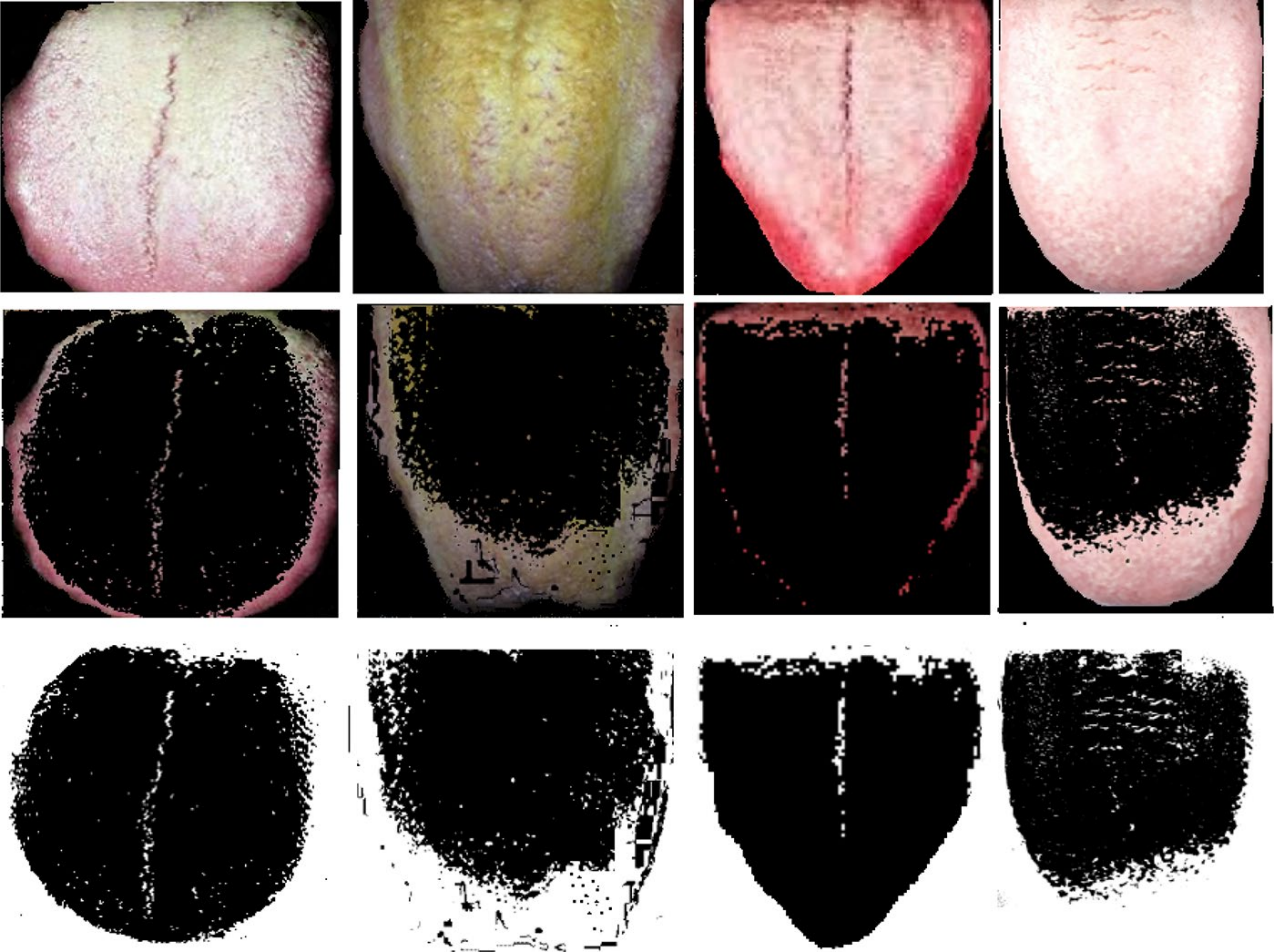
Fur layer extraction of the considered tongue images.

The longitudinal and transverse cracks that groves into the surface of tongue is fissures, if the tongue is fissured and red in color that shows high accumulation of heat, the light fissured tongue indicates the Yin deficiency and the location of the fissure represents an affected organ. If the fissure gets developed in the middle of the tongue, it shows the stomach Yin deficiency; if the sides of the tongue have fissures then the spleen Yin deficiency is reported. The fur is the another feature that has great impact on diabetes, the surface of normal tongue is normally covered with thin white layer, but because of imbalance between Yin-Yang, the layer of tongue appears to be thick fine hair like structure. In Fig. 7, the fur layer is extracted and shown, fur may be white, yellow, black, brown or black, the thickness of fur and color of the fur is considered to be major factor for diabetes diagnosis.

**Figure 8:**
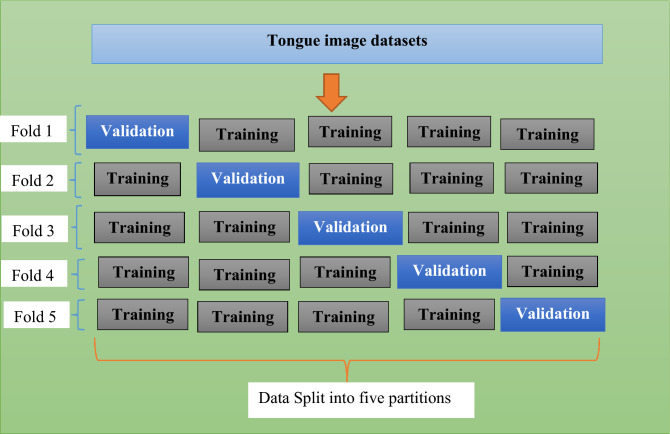
Fivefold cross validation for diagnosis of diabetes mellitus.

In this research paper, fivefold cross-validation is conducted and once 25% data are used for testing, the remaining 25% is equally divided into five equal parts. Hence, the training and validation process are performed in 5 iterations and in each iteration 4 parts are employed for training and 1 part is employed for validation. Figure 8 presents the fivefold cross validation employed for diagnosis of diabetes mellitus.

In the proposed study of 2675 participants, 49.80% of peoples have medium sized tongue, 34.21% peoples have enlarged tongue and 15.98% peoples have small sized tongue. The color of the tongue represents the ‘Qi’ and blood flow in the body, about 52.34% of peoples shows mild red color tongue, 25.30% reported red color, 14.00% have pale colored tongue and 8.30% shows bluish tongue. On investigating the tongue surface, about 55.66% peoples have red spots, 39.02% peoples have black spots, 38.80% peoples shown fissures, and 7.66% of peoples shown petechiae. About 62.14% of participants have tooth markings. About 44.97% of peoples shown white color fur, 7.50% participants have black colored fur, 37.24% peoples have yellow fur. The fur thickness is one of main feature employed for diabetes diagnosis, about 31.85% of participants had thin fur, 60.04% had thick fur and 8.11% had no fur. On analyzing the dryness of the tongue, 72.59% peoples have normal tongue, 19.35% reported dry tongue and 8.05% of peoples reported wet tongue.

The features selected and extracted by the ResNet-50 is passed into the developed Deep RBFNN model for performing classification; the deep RBFNN model is designed with 8 input neurons corresponding to the features such as tongue color, shape, fur color, fur thickness, tongue spots, saliva, fissures, and teeth markings. The hidden layer fixation and hidden neuron fixation is inevitable and significant step in framing of network architecture. The inadequate number of hidden layers and hidden neurons affects the training accuracy, whereas higher their number increase the complexity of the network and cause over-fitting issue so it is necessary to fix optimal number of hidden layers and hidden neurons. Based on trial and error method the hidden layers are fixed to 7 with neurons of 3–5–7–5-3–4–3 in each layer. The output layer has neurons of 2 units that demonstrate whether the input image falls in diabetes class or non-diabetes class. The classification performance is evaluated by the metrics given through Eq. (26) through (31). Table 4 shows the performance metric values of the proposed hybrid CNN—Deep RBFNN learning diabetes classification module.

**Table 4:** Classification performance of the proposed model.

Model	Accuracy	Precision	Sensitivity	Specificity	F1Score	Error rate
ResNet50	0.929	0.961	0.956	0.776	0.958	0.071
ResNet50-RBFNN	0.944	0.968	0.965	0.819	0.967	0.056
ResNet50-DeepRBFNN	0.984	0.989	0.991	0.943	0.990	0.016

The training and testing performance is presented in Table 5, the performance of the proposed model is investigated by employing ResNet-50 for classifying the dataset and it reported classification accuracy of 0.885 for training and 0.929 for testing. To improve the classification performance, the last layer of ‘soft-max’ is removed, the flattened data is fed into the RBFNN model with traditional learning strategy and the classification metric results are reported. Table 6 presents the training and testing mean square error elapsed with respect to the number of epochs.

**Table 5:** Training and Testing Performance of the model.

Model	Training accuracy (%)	Testing accuracy (%)
ResNet50	0.885	0.929
ResNet50-RBFNN	0.923	0.944
ResNet50-Deep RBFNN	0.989	0.984

**Table 6:** Error variations based on number of epochs evolved.

Training variations		Testing variations	
Number of epochs	Mean square error	Number of epochs	Mean square error
10	2.1629	10	3.0128
20	1.4127	20	2.7219
30	1.0054	30	2.2209
40	0.0061	40	1.7286
50	0.00175	50	0.09963
56	0.00034	60	0.01692
The neural model got converged at 56th epoch in the training process		73	0.00721
		The trained neural model got converged at 73rd epoch in the testing process	

**Figure 9:**
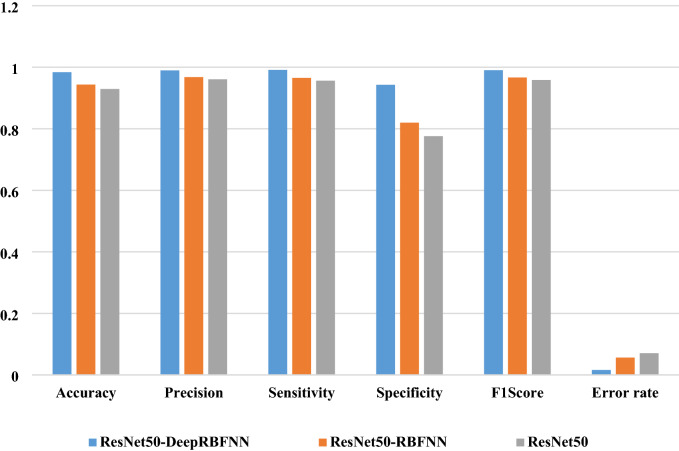
Performance analysis of the proposed diabetes diagnosis in humans.

**Figure 10:**
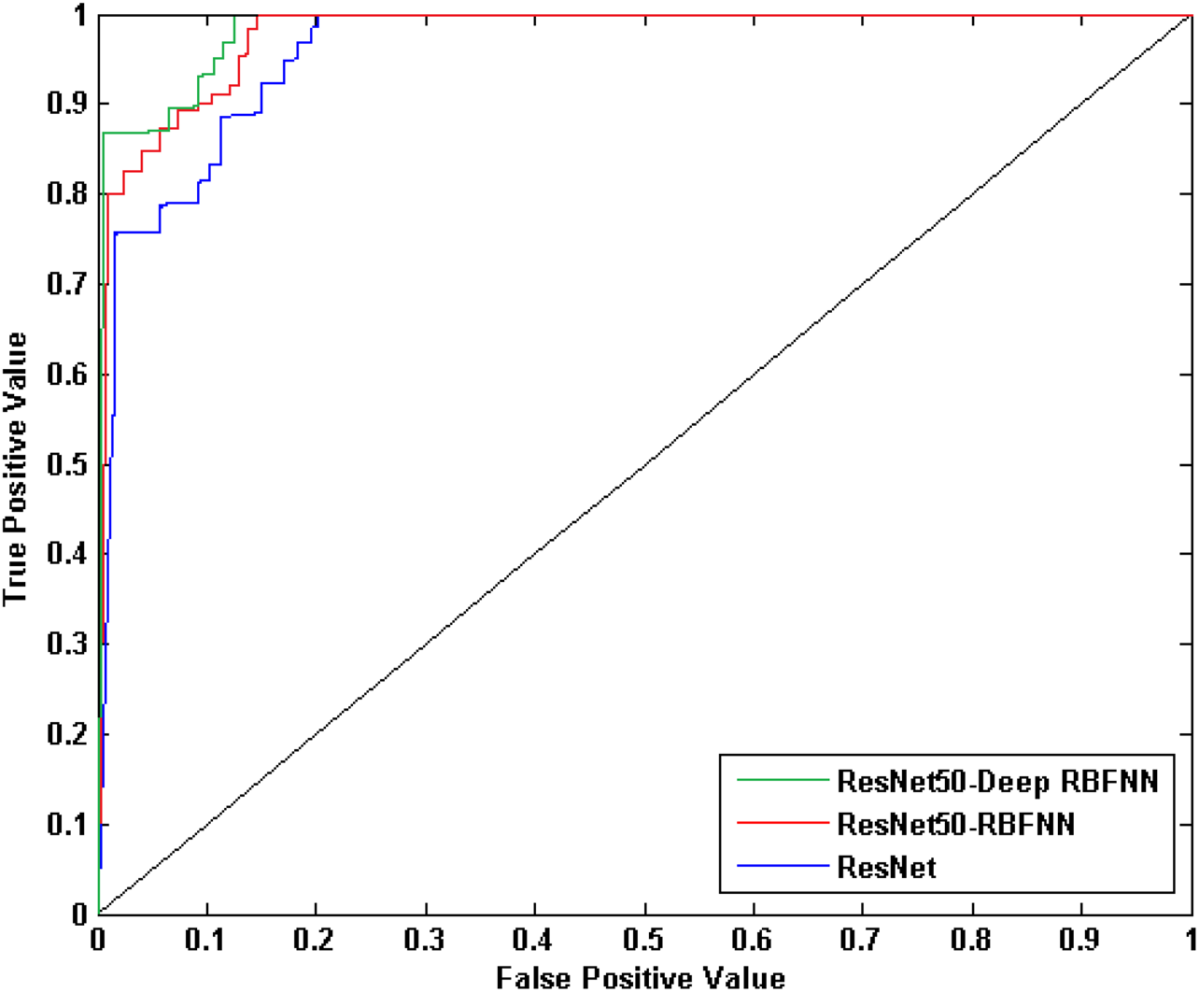
ROC plot for the deep RBFNN diabetes diagnosis model.

On comparing with conventional CNN model, the RBFNN incorporated strategy improved accuracy, true positive prediction rate, true negative prediction rate whereas the error rate is significantly reduced than the strategy when ResNet-50 alone is employed for diabetes diagnosis as depicted in Fig. 9. The training accuracy of ResNet50-RBFNN model is 92.3% and the testing accuracy is 94.4%. To further enhance the model performance deep learning strategy has been employed, here the training is preformed based on auto-encoder based learning algorithm and the model reports significant improvement in diagnosis performance with training accuracy of 98.9% and testing accuracy of 98.4%, the error rate has been reduced with the value of 0.071. The ROC plot depicting the behaviour of the proposed model is shown in Fig. 10.

The performance of the proposed ResNet50-Deep RBFNN for diabetes classification is compared with existing works of literature^17,18,27,29,43–48^, such as ResNet34, AlexNet, SqueezeNet and SVM based strategy and obtained metric results are presented in Table 7. The performance of proposed Deep RBFNN with ResNet50 architecture significantly improved the performance than that of the other deep networks such as AlexNet, SqueezeNet, and ResNet34 models employed for diabetes classification. It is noted that the accuracy of the proposed model is better than that of ResNet 34 with 10.9%, 12.8% than the SqueezeNet and 12.1% than that of AlexNet. The precision of the deep proposed RBFNN model is significantly improved with the value of 9.9% than the ResNet 34, 12.4% than the SqueezeNet and 11.9% than the AlexNet.

**Table 7:** Comparative analysis with previous works.

Classification models	Accuracy	Precision	Sensitivity	Specificity	F1 Score	Error rate	Computational time (min)
PCA- GA-SVM—Zhang et al.^17^	0.670	0.669	0.769	0.555	0.715	0.329	2.01
Non-invasive approach—Zhang ^43^	0.763	0.691	0.728	0.607	0.774	0.317	3.46
Greedy Snake Algorithm—Naveed and Geetha ^44^	0.801	0.760	0.803	0.669	0.792	0.258	3.19
ResNet 34—Wang et al. [^27^]	0.875	0.890	0.907	0.826	0.898	0.125	3.24
SqueezeNet—Wu et al.^29^	0.856	0.865	0.899	0.793	0.882	0.143	3.31
AlexNet—Huo et al.^18^	0.863	0.870	0.905	0.801	0.887	0.137	3.27
Random forest algorithm—Xiang et al.^45^	0.877	0.881	0.893	0.829	0.890	0.133	3.07
Stacking model—Li et al.^46^	0.892	0.894	0.912	0.850	0. 917	0.119	3.61
GA_XGBT approach—Li et al.^47^	0.906	0.911	0.899	0.872	0.934	0.103	3.57
SVM classifiers—Sagayaraj et al.^48^	0.927	0.932	0.945	0.917	0.955	0.085	3.41
Proposed ResNet50-Deep RBFNN model	0.984	0.989	0.991	0.943	0.990	0.016	3.50

The sensitivity employing the proposed ResNet50-Deep RBFNN model is higher than that of ResNet-34 by 8.4%, SqueezeNet by 9.2% and AlexNet by 8.6%. The specificity of the proposed model is better than that of other deep networks with the rate of 11.7% than the ResNet, 15% than the SqueezeNet and 14.2% than of AlexNet. The F1 Score of the Deep RBFNN with Resnet 50 framework is 9.2% better than that of ResNet 34, 10.8% than the SqueezeNet and 10.3% than the AlexNet. The error rate of the proposed model is greatly reduced than the other deep neural nets and the performance is significantly higher than that of SVM based strategy^17^. The computational time of SVM is better than that of deep neural networks, but the classification performance is not to the desired level. So, on considering the deep neural networks the proposed Deep RBFNN model has average computational time of 23 s higher than that of other deep nets, but the metric values is significantly improved than that of other deep networks.

The significance of the proposed diagnosis strategy over other previous models is demonstrated by performing statistical analysis^49,50^. The significance analysis is presented based on $5 \times 2$ CV test, where the entire dataset is segmented into 5 folds and iterated for five times. At each iteration, the training and testing datasets are rotated. Considering two models the performance is compared by,

32 $$ Acc_{A} = Acc_{A,Model - 1} - Acc_{A,Model - 2} $$

33 $$ Acc_{B} = Acc_{B,Model - 1} - Acc_{B,Model - 2} $$

The mean and variance of difference is evaluated,

34 $$ Acc_{avg} = (Acc_{A} + Acc_{B} )/2 $$

35 $$ \sigma^{2} = (Acc_{A} - Acc_{avg} )^{2} + (Acc_{B} - Acc_{avg} )^{2} $$

The ‘*t*’ statistics is evaluated after 5 iterations and is as follows:

36 $$ t = \frac{{Acc_{A,1} }}{{\sqrt {(1/5)\sum\nolimits_{i = 1}^{5} {\sigma_{i}^{2} } } }} $$

where, $^{\prime}Acc_{A,1} ^{\prime}$ indicates the accuracy of first iteration of ‘*A*’ sample.

The ‘*t*’ distribution with 5 degrees of freedom is followed by ‘*t*’ statistics, the *p*-value is estimated and compared with the level of significance $\alpha = 0.05$ in order to illustrate the level of hypothesis and is presented in Table 8. The proposed ResNet-50 Deep RBFNN model has null hypothesis rejection with ResNet50 and ResNet50-RBFNN models proving its better classification performance.

**Table 8:** Significance test with level of significance 0.05.

Models under comparison	p-value	Statistical result
ResNet50- Deep RBFNN vs ResNet 50	0.0347	Null hypothesis rejection
ResNet50-Deep RBFNN vs ResNet50-RBFNN	0.0421	Null hypothesis rejection

## Conclusion

In this research study, a non-invasive technique of diabetes diagnosis based on TCM was developed by employing deep auto encoder learning algorithm with CNN networks. The tongue being the index of internal organs, its characteristics such as texture, geometry, and color depicts the balance that exists between the five element theory and Yin-Yang concept of TCM. The deep features of panoramic tongue images were extracted by CNN-ResNet 50 architecture and the extracted features are trained with Deep RBFNN classifier model. The performance of the proposed model is compared with other existing works of literature such as ResNet 34, AlexNet, SqueezeNet and it was investigated that the proposed deep RBFNN model has shown improved performance with better classification accuracy and reduced error rate. The significance of the proposed model as well was analysed based on $5 \times 2$ CV test and it was demonstrated that the proposed model statistically fits to perform diabetes diagnosis.
