# Author Correction: Panoramic tongue imaging and deep convolutional machine learning model for diabetes diagnosis in humans

**DOI:** 10.1038/s41598-022-23610-1

**Published:** 2022-11-09

**Authors:** Saritha Balasubramaniyan, Vijay Jeyakumar, Deepa Subramaniam Nachimuthu

**Affiliations:** 1 Department of Electronics and Communication Engineering, Jai Shriram Engineering College, Tiruppur, Tamil Nadu 638 660 India; 2 Department of Biomedical Engineering, Sri Sivasubramaniya Nadar College of Engineering, Chennai, Tamil Nadu 603 110 India; 3 Department of Electrical Engineering, National Institute of Technology Arunachal Pradesh, YupiaPapum Pare District, Arunachal Pradesh 791112 India

Correction to: *Scientific Reports* https://doi.org/10.1038/s41598-021-03879-4, published online 07 January 2022

In the original version of this Article, Deepa Subramaniam Nachimuthu was incorrectly listed as a corresponding author. The correct corresponding author for this Article is Saritha Balasubramaniyan. Correspondence and requests for materials should be addressed to sarithabme89@gmail.com.

The original Article has been corrected.

